# Pi sampling: a methodical and flexible approach to initial macromolecular crystallization screening

**DOI:** 10.1107/S0907444911008754

**Published:** 2011-04-07

**Authors:** Fabrice Gorrec, Colin M. Palmer, Guillaume Lebon, Tony Warne

**Affiliations:** a MRC Laboratory of Molecular Biology, Hills Road, Cambridge CB2 0QH, England

**Keywords:** macromolecular crystallization, initial screen formulation, incomplete factorial approach, modular distribution, membrane-protein crystallization, GPCR

## Abstract

The Pi sampling method is derived from the incomplete factorial approach to macromolecular crystallization screen design. The resulting ‘Pi screens’ have a modular distribution of a given set of up to 36 stock solutions. Maximally diverse conditions can be produced by taking into account the properties of the chemicals used in the formulation and the concentrations of the corresponding solutions. The Pi sampling method has been implemented in a web-based application that generates screen formulations and recipes. It is particularly adapted to screens consisting of 96 different conditions. The flexibility and efficiency of Pi sampling is demonstrated by the crystallization of soluble proteins and of an integral membrane-protein sample.

## Abstract 2

Pi sampling, derived from the incomplete factorial approach, is an effort to maximize the diversity of macromolecular crystallization conditions and to facilitate the preparation of 96-condition initial screens.

## 1. Introduction   

A crucial aspect of macromolecular crystallographic studies is finding suitable conditions for the crystallization of a sample. This can be difficult because many factors alter the crystallization behaviour of macromolecules, including the type and the concentration of the chemicals employed to formulate the conditions (McPherson, 1990 ▶). A condition includes at least a precipitant and most conditions also include a buffer and an additive. During the initial crystallization experiments, the structure of the macromolecule is not known and hence the most efficient formulation cannot be predicted. As a consequence, one should be cautious when making initial assumptions and limiting choices in subsequent optimizations (Rupp, 2003 ▶). Nonetheless, the number of initial crystallization con­ditions cannot be unreasonably large since purified protein is often difficult and expensive to produce in large quantities.

There are essentially two approaches to restrict an initial screen to a limited number of crystallization conditions. Firstly, a sparse-matrix formulation can be used, which consists of an empirically derived combination of components based on known or published crystallization conditions (Jancarik & Kim, 1991 ▶). Secondly, an incomplete factorial formulation can be generated in which selected components are combined to prepare new conditions in accordance with principles of randomization and balance (Carter & Carter, 1979 ▶). Numerous commercial screens based on these two main approaches are available. Automated systems have been implemented at the Medical Research Council (MRC) Laboratory of Molecular Biology (LMB) to test these as routine initial screens using the 96-well crystallization plate format (Stock *et al.*, 2005 ▶). However, for various reasons, many laboratories opt for a minimal screen (Kimber *et al.*, 2003 ▶) and still perform at least some aspects of the work manually (Bergfors, 2007 ▶).

Here, we present a development based on the incomplete factorial formulation: the Pi sampling method. The name of the method was inspired by the story of Archimedes, who used the ‘method of exhaustion’ (*i.e.* an empirical approach) with a 96-sided polygon in order to reach the first good numerical approximation of π (Smith, 1958 ▶). Pi sampling uses modular arithmetic to form combinations of three stock solutions across a 96-condition grid. Maximally diverse conditions can be produced by taking into account the properties of the chemicals used in the formulation and the concentrations of the corresponding stock solutions. We have implemented this approach in a web-based application called *Pi Sampler*: user input consists of the details of up to 36 stock solutions, from which the application generates the formulations for a 96-­condition screen. The Pi sampling method is intended to help laboratories to test new crystallization-screen formulations on a day-to-day basis based on the properties of the macromolecules investigated, as has been performed pre­viously with RNA (Doudna *et al.*, 1993 ▶).

Firstly, we tested Pi sampling with ten commercially available soluble proteins. For this, the ‘Pi minimal screen’ was employed including a wide variety of well known chemicals frequently used for macromolecular crystallization.

We then investigated the impact of Pi sampling on the crystallization of a G-protein-coupled receptor (GPCR) that had been difficult to crystallize: the adenosine A_2A_ receptor (construct A_2A_R-GL31). We formulated another Pi screen, the ‘Pi-PEG screen’, taking into consideration general observations made about crystallization of integral membrane-protein samples. Previous crystallization experiments on another GPCR (the β_1_-adrenergic receptor) had indicated that the use of simple proprietary screens formulated with poly(ethylene glycol) (PEG) and buffers gave a greater yield of crystals than all commercially available screens, including those geared towards membrane proteins (Warne *et al.*, 2009 ▶), and the 2.7 Å resolution structure was solved using conditions optimized from a proprietary screen essentially based on PEGs (Warne *et al.*, 2008 ▶). This has been observed previously with other membrane-protein targets (Lemieux *et al.*, 2003 ▶). In addition, mixtures of polyethylene glycols have been used successfully to develop a minimal screen (Brzozowski & Walton, 2001 ▶) and to study crystal structures of the Kir potassium channel (Clarke *et al.*, 2010 ▶). Such mixtures were incorporated into the Pi-PEG screen.

## 2. Methods   

### 2.1. Pi sampling   

Pi sampling begins with up to 36 stock solutions, divided into three sets of 12. The first set of solutions is used in the screen at constant concentration. The second and third sets are added according to a gradient between specified minimum and maximum concentrations. Typically, the first set is com­posed of buffers and the second and third sets are precipitants/additives.

**Figure 1:**
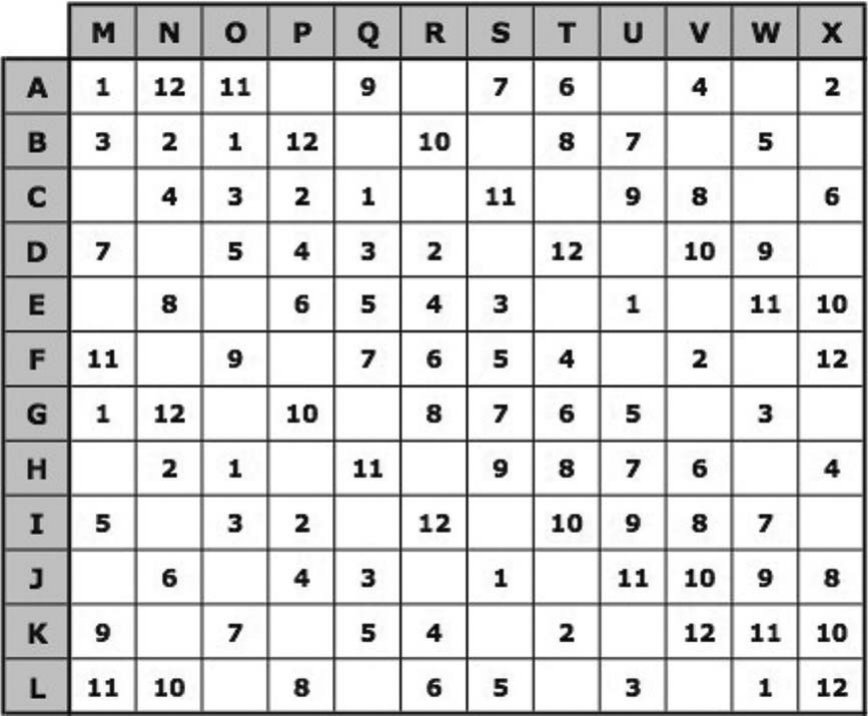
Pi sampling: combinations of stock solutions from three different sets (see also http://pisampler.mrc-lmb.cam.ac.uk/).

The combinations of three stock solutions (one from each set) are generated according to Fig. 1 ▶, where 1–12 refer to the IDs for solutions of the first set, A–M to those of the second set and N–X to those of the third set. The number in each cell shows which solution of the first set will be combined with the corresponding solutions of the second and third sets. Blank spaces show when no such combinations are generated.

**Figure 2:**
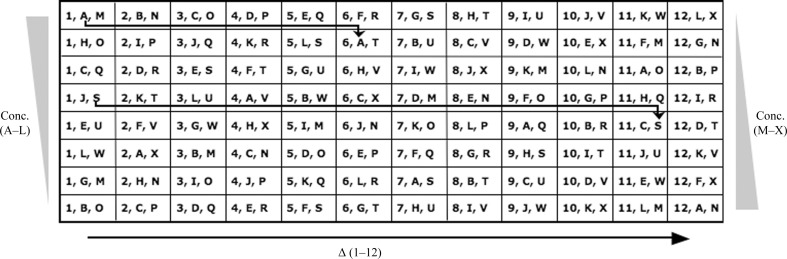
Pi sampling: combinations of the stock solutions in a 96-condition plate layout (well A1 is at the top left corner). Each solution of set 1 (ID 1–12) is seen in the eight conditions forming a column of the plate. The Δ values of set 1 increase from left to right in the screen layout. The positions of the solutions A–L (set 2) shift across five columns and down one row (Δ values not represented). The positions of solutions M–X (set 3) shift across ten columns and down one row. Gradients of concentration for sets 2 and 3 are represented on the left and right, respectively.

Fig. 2 ▶ summarizes the distribution of the stock solutions in a standard 96-condition plate layout (*i.e.* 12 columns and eight rows).

Set 1: each solution (ID 1–12) is seen in the eight conditions forming a column of the plate. A variable Δ should be associated with the stock solutions. The variable Δ corresponds to a property of the solution selected (*e.g.* pH, molecular weight of the main chemical, absorption properties or others). Δ values increase from left to right in the screen layout.
Set 2: each solution (ID A–L) is represented once in each row. The final concentrations decrease gradually from the top to the bottom of the screen layout, forming a gradient. The distribution of solutions A–L is based on the sequence of Δ values established for set 1: the positions of the solutions shift across five columns and down one row. Solutions A–L should also be associated with a variable Δ and hence a sequence is formed for the distribution of the third set of solutions.
Set 3: each solution (ID M–X) is also represented once in each row. The final concentrations increase gradually from the top to the bottom of the screen, forming another gradient. The solutions M–X are distributed with the same modulo arithmetic operation as previously, but with respect to the Δ values of solutions A–L. For example, solution M is mixed with solution A in the first row, solution F in the second row, solution K in the third row and so on, as shown in Fig. 2 ▶. This means that both the second and third sets are arranged according to the same modulo arithmetic operation (5 modulo 12); however, when looking at the plate layout, the positions of solutions M–X shift across ten columns and down one row.

### 2.2. *Pi Sampler*    

*Pi Sampler* can be accessed *via* the internet at http://pisampler.mrc-lmb.cam.ac.uk/. Users can enter the details of up to 36 stock solutions, including stock concentrations, desired screen concentration ranges and Δ values. The application then generates a 96-condition screen formulation following the Pi sampling method described above. Formulations, recipes and total required volumes of stock solutions are presented and may conveniently be downloaded in comma-separated variable format (CSV), allowing the user to import them into other software for automated screen making (Cox & Weber, 1987 ▶), formulation analysis (Hedderich *et al.*, 2011 ▶) and data mining (Kantardjieff & Rupp, 2004 ▶). The parameters used to generate the screen can also be saved and uploaded in the same format. Further details and instructions can be found on the website.

### 2.3. Pi minimal screen preparation and crystallization assays with commercially available soluble proteins   

The final formulation of the Pi minimal screen can be found in Table 1 ▶. There are 36 starting stock solutions overall. Each solution composing the first set (ID 1–12) is a mixture of an acid with its corresponding base (*e.g.* HEPES pH 7.5: 1 *M* HEPES solution mixed with 1 *M* HEPES sodium salt in order to reach pH 7.5), except for buffer 11 (AMPD mixed with Tris base). Note that this is also true for the precipitant phosphate (phosphate system: sodium dihydrogen phosphate/dipotassium hydrogen phosphate). Values of pH (4.0–9.5) were chosen as the variable Δ for the first set, whilst arbitrary values were chosen for additives of various natures composing the second set (ID A–L). Eventually, a few conditions were made without additive/buffer because of chemical incompatibilities (Table 1 ▶).

Highest purity grade chemicals (Molecular Biology grade when available) were purchased from Sigma–Aldrich to prepare 36 stock solutions. The solutions were mixed in 96 Falcon tubes. The screen was dispensed into ‘MRC original plates’ (96-well, two-drop, Swissci; Stock *et al.*, 2005 ▶).

Commercial proteins that had been crystallized before were chosen to prepare test samples. Protein concentrations were chosen randomly between 7 and 150 mg ml^−1^ (Table 2 ▶). Vapour-diffusion experiments were set up at 295 K, mixing two different sample: condition ratios (1:3 and 3:1) to give a final volume of 400 nl. The plates were then stored at 291 K. A condition was considered to be a hit when at least one of the two corresponding drops con­tained crystals with well known morphology after one week. Table 3 ▶ shows the ‘hits per con­dition’ observed and the corresponding results expected for the binomial distribution (see §[Sec sec4.2]4.2).

### 2.4. Pi-PEG screen preparation and crystallization assays with a GPCR   

The final formulation of the Pi-PEG screen can be found in Table 4 ▶. The formulation can also be generated using *Pi Sampler* by loading the Pi-PEG example data. The pH values (4.8–8.8) were chosen as the variable Δ for the buffers composing set 1 (ID 1–12), whilst molecular weight was chosen for set 2 (PEGs A–L, final concentration range 0–22.5%). The same 12 PEGs were used for set 3 (PEGs M–X, final concentration range 0–45%). General details of the preparation are similar to §[Sec sec2.3]2.3, but there are 24 stock solutions at the start (instead of 36). Vapour-diffusion experiments were set up at 277 K, mixing sample and condition in a 1:1 ratio to give a final volume of 200 nl. The preparation of A_2A_R-GL31 will be published elsewhere (Lebon *et al.*, submitted work). Crystal X-ray screening was performed at the Diamond synchrotron light source (microfocus beamline I24 equipped with a Pilatus 6M detector).

## 3. Results   

There were 116 crystallization hits overall for the experiments with the Pi minimal screen (Table 2 ▶). Some conditions produced hits for several samples (Table 3 ▶).

**Figure 3:**
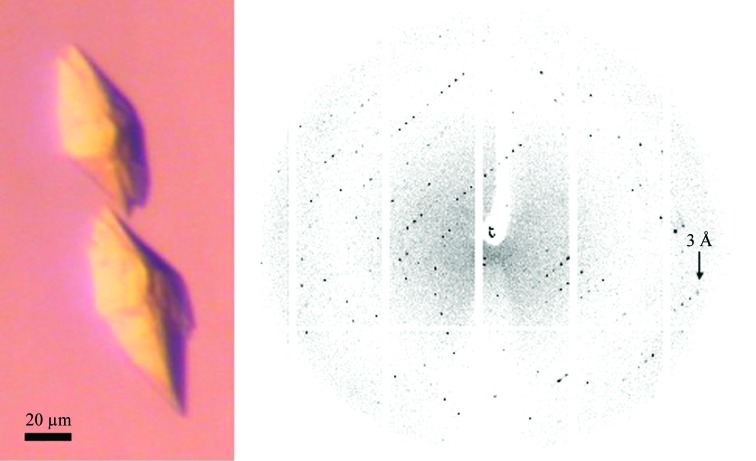
Crystals of A_2A_R-GL31 obtained with the Pi-PEG screen (Table 4 ▶) and an example of a corresponding diffraction pattern.

The Pi-PEG screen yielded crystals that diffracted to 3.0 Å resolution for A_2A_R-GL31 with bound agonist. Fig. 3 ▶ shows the crystals of A_2A_R-GL31 obtained in well E9 [50 m*M* Tris–HCl pH 7.6, 9.6%(*v*/*v*) PEG 200, 22.9%(*v*/*v*) PEG 300] and an example of the corresponding diffraction pattern (no cryoprotectant was required).

## 4. Discussion   

### 4.1. Pi sampling   

In order to understand the rationale behind the modular arithmetic employed for the Pi sampling, it may help to imagine, on a 12 h clock, a series of events occurring every 5 h. The first event is at noon, the second at 5 pm, then 10 pm, then 3 am *etc*. Eventually, there is a succession of 12 events occurring at different hours, with as much time as possible in between each event. If we now look at combinations of three components, there are originally 12^3^ or 1728 possibilities. *Pi Sampler* generates 96 of these combinations that correspond to conditions that are distant in properties. The variety between conditions is then accentuated using a number of different concentrations of solutions (Fig. 2 ▶). If the first and second sets of solutions are ordered according to physico-chemical properties, the generated screen will be an incomplete factorial sampling of interactions between chemicals with these properties. If the chemicals selected have completely different natures, they can be arranged randomly (see §[Sec sec2.3]2.3). The ordering of the third set of solutions can be used to avoid obvious chemical incompatibilities (*e.g.* mixing phosphate and magnesium salts). It is also possible to design simpler screens with only two sets of stock solutions.

### 4.2. The Pi minimal screen   

In order to check the homogeneity of the hits across the screen with the ten samples, we compared the results obtained with what would be expected if each condition had the same probability of hits overall (Table 3 ▶). This can be approximated by a binomial distribution. The probability of success for the binomial distribution is the observed probability for ten attempts: 116/(10 × 96) = 0.12083. The χ^2^ statistic for the data is 3.48. This can be compared with the quantiles of a χ^2^ distribution with two degrees of freedom, which gives a *p* value of 0.18 (calculations not shown). This χ^2^ test indicates that no conditions are obvious outliers with regard to success or failure. There are, however, a multitude of possible biases implied when proceeding with crystallization experiments (which would be even more accentuated with the use of novel samples); hence, any statistical analysis should be taken with precaution. Nonetheless, it is interesting to see that the analysis of the distribution is in accordance with the original approach based on balanced randomization (Carter & Carter, 1979 ▶; Rupp, 2003 ▶).

In addition, the conditions of the Pi minimal screen show no identities to the extensive list of conditions (7230) from commercial screens stored in the ‘PICKScreens’ database (Hedderich *et al.*, 2011 ▶).

### 4.3. The Pi-PEG screen   

The extent of effects on crystallization for precipitants such as PEGs is correlated with their concentrations (McPherson, 1976 ▶) and molecular weights (Forsythe *et al.*, 2002 ▶). The Pi-PEG screen covers a wide range of parameters (kinetics of equilibrium, protein stabilization *etc*.). In addition, the con­centrations of the two different PEGs in a condition can be adjusted for condition optimization (Stock *et al.*, 2005 ▶) and for crystal cryoprotection (Berejnov *et al.*, 2006 ▶). Furthermore, the PICKScreens database shows that the Pi-PEG screen is unique (as for the Pi minimal screen; see §[Sec sec4.2]4.2).

Samples of A_2A_R-GL31 purified in a number of different detergents rarely crystallized in commercially available screens used at the LMB (Stock *et al.*, 2005 ▶) and when they did the crystal quality was not sufficient for structure determination. The first quality crystals were recently obtained using the Pi-PEG screen.

## 5. Conclusions   

We have demonstrated that the Pi sampling is a methodical and flexible approach to initial screening for macromolecular crystallization. Two unique screens produced *de novo* have resulted from this strategy. The Pi minimal screen potentially has an ideal formulation for crystallization of novel soluble protein samples. The Pi-PEG screen is a tailor-made screen for GPCRs and potentially other membrane proteins generated by biasing the formulation towards components known to be essential.

Further screens can be formulated with the *Pi Sampler* on a day-to-day basis in order to test chemicals and techniques, with the aim of increasing the yield of quality crystals. Also, new crystallization techniques are constantly emerging for macromolecular targets such as membrane proteins and hence formulations with special considerations are required: one may want to formulate screens compatible with the lipidic cubic phase (LCP) concept (Landau & Rosenbusch, 1996 ▶) or make extensive use of detergents (Koszelak-Rosenblum *et al.*, 2009 ▶).

In order for laboratories to be able to handle many Pi screen formulations and the flow of resulting data, we are working on the integration of *Pi Sampler* into the ‘xtalPiMS’ Laboratory Information Management System (LIMS; Morris *et al.*, 2011 ▶; see http://www.pims-lims.org).

## Figures and Tables

**Table 1:** Final formulation of the Pi minimal screen ADA, *N*-(2-acetamido)iminodiacetic acid; AMPD, 2-amino-2-methyl-1,3-propanediol; CAPSO, 3-(cyclohexylamino)-2-hydroxy-1-propanesulfonic acid; HEPES, 4-(2-hydroxyethyl)piperazine-1-ethanesulfonic acid; MOPS, 3-(*N*-morpholino)propanesulfonic acid; PEG, poly(ethylene glycol); TAPS, *N*-[Tris(hydroxymethyl)methyl]-3-aminopropanesulfonic acid.

	Set 1				Set 2				Set 3			
Well	ID	Name	Conc.	Unit	ID	Name	Conc.	Unit	ID	Name	Conc.	Unit
A1	1	Formate pH 4.0	0.15	*M*	A	Potassium bromide	0.160	*M*	M	Phosphate	0.6	*M*
A2	2	Acetate pH 4.5	0.15	*M*	B	PEG 300	8.000	%(*v*/*v*)	N	PEG MME 550	24.00	%(*v*/*v*)
A3	3	Malate pH 5.0	0.15	*M*	C	Magnesium sulfate	0.160	*M*	O	Ammonium nitrate	2.0	*M*
A4	4	Citrate pH 5.5	0.15	*M*	D	Sodium fluoride	0.032	*M*	P	PEG 20000	10.0	%(*w*/*v*)
A5	5	MES pH 6.0	0.15	*M*	E	Potassium thiocyanate	0.080	*M*	Q	PEG 1000	30.0	%(*w*/*v*)
A6	6	Cacodylate pH 6.5	0.15	*M*	F	Sodium iodide	0.160	*M*	R	Sodium chloride	1.6	*M*
A7	7	MOPS pH 7.0	0.15	*M*	G	Propanediol	8.000	%(*v*/*v*)	S	PEG 4000	24.0	%(*w*/*v*)
A8	8	HEPES pH 7.5	0.15	*M*	H				T	Lithium sulfate	0.8	*M*
A9	9	Tris pH 8.0	0.15	*M*	I	Ethylene glycol	8.000	%(*v*/*v*)	U	PEG MME 5000	20.0	%(*w*/*v*)
A10	10	TAPS pH 8.5	0.15	*M*	J	Sodium potassium tartrate	0.080	*M*	V	Glycerol	36.0	%(*w*/*v*)
A11	11	AMPD/Tris pH 9.0	0.15	*M*	K	MPD	8.000	%(*v*/*v*)	W	Ammonium sulfate	1.4	*M*
A12	12	CAPSO pH 9.5	0.15	*M*	L	2-Butanol	8.000	%(*v*/*v*)	X	PEG 8000	20.0	%(*w*/*v*)
B1	1	Formate pH 4.0	0.15	*M*	H	Calcium chloride	0.070	*M*	O	Ammonium nitrate	2.3	*M*
B2	2	Acetate pH 4.5	0.15	*M*	I	Ethylene glycol	7.000	%(*v*/*v*)	P	PEG 20000	12.0	%(*w*/*v*)
B3	3	Malate pH 5.0	0.15	*M*	J	Sodium potassium tartrate	0.070	*M*	Q	PEG 1000	35.0	%(*w*/*v*)
B4	4	Citrate pH 5.5	0.15	*M*	K	MPD	7.000	%(*v*/*v*)	R	Sodium chloride	1.8	*M*
B5	5	MES pH 6.0	0.15	*M*	L	2-Butanol	7.000	%(*v*/*v*)	S	PEG 4000	28.0	%(*w*/*v*)
B6	6	Cacodylate pH 6.5	0.15	*M*	A	Potassium bromide	0.140	*M*	T	Lithium sulfate	0.9	*M*
B7	7	MOPS pH 7.0	0.15	*M*	B	PEG 300	7.000	%(*v*/*v*)	U	PEG MME 5000	23.0	%(*w*/*v*)
B8	8	HEPES pH 7.5	0.15	*M*	C	Magnesium sulfate	0.140	*M*	V	Glycerol	42.0	%(*w*/*v*)
B9	9	Tris pH 8.0	0.15	*M*	D	Sodium fluoride	0.028	*M*	W	Ammonium sulfate	1.6	*M*
B10	10	TAPS pH 8.5	0.15	*M*	E	Potassium thiocyanate	0.070	*M*	X	PEG 8000	23.0	%(*w*/*v*)
B11	11	AMPD/Tris pH 9.0	0.15	*M*	F	Sodium iodide	0.140	*M*	M	Phosphate	0.7	*M*
B12	12	CAPSO pH 9.5	0.15	*M*	G	Propanediol	7.000	%(*v*/*v*)	N	PEG MME 550	28.00	%(*v*/*v*)
C1	1	Formate pH 4.0	0.15	*M*	C	Magnesium sulfate	0.120	*M*	Q	PEG 1000	39.0	%(*w*/*v*)
C2	2	Acetate pH 4.5	0.15	*M*	D	Sodium fluoride	0.024	*M*	R	Sodium chloride	2.1	*M*
C3	3	Malate pH 5.0	0.15	*M*	E	Potassium thiocyanate	0.060	*M*	S	PEG 4000	31.0	%(*w*/*v*)
C4	4	Citrate pH 5.5	0.15	*M*	F	Sodium iodide	0.120	*M*	T	Lithium sulfate	1.0	*M*
C5	5	MES pH 6.0	0.15	*M*	G	Propanediol	6.000	%(*v*/*v*)	U	PEG MME 5000	26.0	%(*w*/*v*)
C6	6	Cacodylate pH 6.5	0.15	*M*	H	Calcium chloride	0.060	*M*	V	Glycerol	47.0	%(*w*/*v*)
C7	7	MOPS pH 7.0	0.15	*M*	I	Ethylene glycol	6.000	%(*v*/*v*)	W	Ammonium sulfate	1.8	*M*
C8	8	HEPES pH 7.5	0.15	*M*	J	Sodium potassium tartrate	0.060	*M*	X	PEG 8000	26.0	%(*w*/*v*)
C9	9	Tris pH 8.0	0.15	*M*	K	MPD	6.000	%(*v*/*v*)	M	Phosphate	0.8	*M*
C10	10	TAPS pH 8.5	0.15	*M*	L	2-Butanol	6.000	%(*v*/*v*)	N	PEG MME 550	31.00	%(*v*/*v*)
C11	11	AMPD/Tris pH 9.0	0.15	*M*	A	Potassium bromide	0.120	*M*	O	Ammonium nitrate	2.6	*M*
C12	12	CAPSO pH 9.5	0.15	*M*	B	PEG 300	6.000	%(*v*/*v*)	P	PEG 20000	13.0	%(*w*/*v*)
D1	1	Formate pH 4.0	0.15	*M*	J				S	PEG 4000	35.0	%(*w*/*v*)
D2	2	Acetate pH 4.5	0.15	*M*	K	MPD	5.000	%(*v*/*v*)	T	Lithium sulfate	1.1	*M*
D3	3	Malate pH 5.0	0.15	*M*	L	2-Butanol	5.000	%(*v*/*v*)	U	PEG MME 5000	38.00	%(*v*/*v*)
D4	4	Citrate pH 5.5	0.15	*M*	A	Potassium bromide	0.100	*M*	V	Glycerol	52.0	%(*w*/*v*)
D5	5	MES pH 6.0	0.15	*M*	B	PEG 300	5.000	%(*v*/*v*)	W	Ammonium sulfate	2.0	*M*
D6	6	Cacodylate pH 6.5	0.15	*M*	C	Magnesium sulfate	0.100	*M*	X	PEG 8000	29.0	%(*w*/*v*)
D7	7	MOPS pH 7.0	0.15	*M*	D	Sodium fluoride	0.020	*M*	M	Phosphate	0.9	*M*
D8	8	HEPES pH 7.5	0.15	*M*	E	Potassium thiocyanate	0.050	*M*	N	PEG MME 550	34.00	%(*v*/*v*)
D9	9	Tris pH 8.0	0.15	*M*	F	Sodium iodide	0.100	*M*	O	Ammonium nitrate	2.9	*M*
D10	10	TAPS pH 8.5	0.15	*M*	G	Propanediol	5.000	%(*v*/*v*)	P	PEG 20000	15.0	%(*w*/*v*)
D11	11				H	Calcium chloride	0.050	*M*	Q	PEG 1000	43.0	%(*w*/*v*)
D12	12	CAPSO pH 9.5	0.15	*M*	I	Ethylene glycol	5.000	%(*v*/*v*)	R	Sodium chloride	2.3	*M*
E1	1	Formate pH 4.0	0.15	*M*	E	Potassium thiocyanate	0.040	*M*	U	PEG MME 5000	32.0	%(*w*/*v*)
E2	2	Acetate pH 4.5	0.15	*M*	F	Sodium iodide	0.080	*M*	V	Glycerol	57.0	%(*w*/*v*)
E3	3	Malate pH 5.0	0.15	*M*	G	Propanediol	4.000	%(*v*/*v*)	W	Ammonium sulfate	2.2	*M*
E4	4	Citrate pH 5.5	0.15	*M*	H				X	PEG 8000	32.0	%(*w*/*v*)
E5	5	MES pH 6.0	0.15	*M*	I	Ethylene glycol	4.000	%(*v*/*v*)	M	Phosphate	0.9	*M*
E6	6	Cacodylate pH 6.5	0.15	*M*	J	Sodium potassium tartrate	0.040	*M*	N	PEG MME 550	38.0	%(*v*/*v*)
E7	7	MOPS pH 7.0	0.15	*M*	K	MPD	4.000	%(*v*/*v*)	O	Ammonium nitrate	3.1	*M*
E8	8	HEPES pH 7.5	0.15	*M*	L	2-Butanol	4.000	%(*v*/*v*)	P	PEG 20000	16.0	%(*w*/*v*)
E9	9	Tris pH 8.0	0.15	*M*	A	Potassium bromide	0.080	*M*	Q	PEG 1000	48.0	%(*w*/*v*)
E10	10	TAPS pH 8.5	0.15	*M*	B	PEG 300	4.000	%(*v*/*v*)	R	Sodium chloride	2.5	*M*
E11	11	AMPD/Tris pH 9.0	0.15	*M*	C	Magnesium sulfate	0.080	*M*	S	PEG 4000	38.0	%(*w*/*v*)
E12	12	CAPSO pH 9.5	0.15	*M*	D				T	Lithium sulfate	1.3	*M*
F1	1	Formate pH 4.0	0.15	*M*	L	2-Butanol	3.000	%(*v*/*v*)	W	Ammonium sulfate	2.4	*M*
F2	2	Acetate pH 4.5	0.15	*M*	A	Potassium bromide	0.060	*M*	X	PEG 8000	35.0	%(*w*/*v*)
F3	3	Malate pH 5.0	0.15	*M*	B	PEG 300	3.000	%(*v*/*v*)	M	Phosphate	1.0	*M*
F4	4				C	Magnesium sulfate	0.06	*M*	N	PEG MME 550	42.00	%(*v*/*v*)
F5	5	MES pH 6.0	0.15	*M*	D	Sodium fluoride	0.012	*M*	O	Ammonium nitrate	3.4	*M*
F6	6	Cacodylate pH 6.5	0.15	*M*	E	Potassium thiocyanate	0.030	*M*	P	PEG 20000	18.0	%(*w*/*v*)
F7	7	MOPS pH 7.0	0.15	*M*	F	Sodium iodide	0.060	*M*	Q	PEG 1000	52.0	%(*w*/*v*)
F8	8	HEPES pH 7.5	0.15	*M*	G	Propanediol	3.000	%(*v*/*v*)	R	Sodium chloride	2.7	*M*
F9	9	Tris pH 8.0	0.15	*M*	H	Calcium chloride	0.030	*M*	S	PEG 4000	42.0	%(*w*/*v*)
F10	10	TAPS pH 8.5	0.15	*M*	I	Ethylene glycol	3.000	%(*v*/*v*)	T	Lithium sulfate	1.4	*M*
F11	11	AMPD/Tris pH 9.0	0.15	*M*	J	Sodium potassium tartrate	0.030	*M*	U	PEG MME 5000	35.0	%(*w*/*v*)
F12	12	CAPSO pH 9.5	0.15	*M*	K	MPD	3.000	%(*v*/*v*)	V	Glycerol	62.0	%(*w*/*v*)
G1	1	Formate pH 4.0	0.15	*M*	G	Propanediol	2.000	%(*v*/*v*)	M	Phosphate	1.1	*M*
G2	2	Acetate pH 4.5	0.15	*M*	H				N	PEG MME 550	45.00	%(*v*/*v*)
G3	3	Malate pH 5.0	0.15	*M*	I	Ethylene glycol	2.000	%(*v*/*v*)	O	Ammonium nitrate	3.7	*M*
G4	4	Citrate pH 5.5	0.15	*M*	J	Sodium potassium tartrate	0.020	*M*	P	PEG 20000	19.0	%(*w*/*v*)
G5	5	MES pH 6.0	0.15	*M*	K	MPD	2.000	%(*v*/*v*)	Q	PEG 1000	56.0	%(*w*/*v*)
G6	6	Cacodylate pH 6.5	0.15	*M*	L	2-Butanol	2.000	%(*v*/*v*)	R	Sodium chloride	3.0	*M*
G7	7	MOPS pH 7.0	0.15	*M*	A	Potassium bromide	0.040	*M*	S	PEG 4000	45.0	%(*w*/*v*)
G8	8	HEPES pH 7.5	0.15	*M*	B	PEG 300	2.000	%(*v*/*v*)	T	Lithium sulfate	1.5	*M*
G9	9	Tris pH 8.0	0.15	*M*	C	Magnesium sulfate	0.040	*M*	U	PEG MME 5000	38.0	%(*w*/*v*)
G10	10	TAPS pH 8.5	0.15	*M*	D	Sodium fluoride	0.008	*M*	V	Glycerol	67.0	%(*w*/*v*)
G11	11	AMPD/Tris pH 9.0	0.15	*M*	E	Potassium thiocyanate	0.020	*M*	W	Ammonium sulfate	2.6	*M*
G12	12	CAPSO pH 9.5	0.15	*M*	F	Sodium iodide	0.040	*M*	X	PEG 8000	38.0	%(*w*/*v*)
H1	1	Formate pH 4.0	0.15	*M*	B	PEG 300	1.000	%(*v*/*v*)	O	Ammonium nitrate	4.0	*M*
H2	2	Acetate pH 4.5	0.15	*M*	C	Magnesium sulfate	0.020	*M*	P	PEG 20000	20.0	%(*w*/*v*)
H3	3	Malate pH 5.0	0.15	*M*	D	Sodium fluoride	0.004	*M*	Q	PEG 1000	60.0	%(*w*/*v*)
H4	4	Citrate pH 5.5	0.15	*M*	E	Potassium thiocyanate	0.010	*M*	R	Sodium chloride	3.2	*M*
H5	5	MES pH 6.0	0.15	*M*	F	Sodium iodide	0.020	*M*	S	PEG 4000	48.0	%(*w*/*v*)
H6	6	Cacodylate pH 6.5	0.15	*M*	G	Propanediol	1.000	%(*v*/*v*)	T	Lithium sulfate	1.6	*M*
H7	7	MOPS pH 7.0	0.15	*M*	H	Calcium chloride	0.010	*M*	U	PEG MME 5000	40.0	%(*w*/*v*)
H8	8	HEPES pH 7.5	0.15	*M*	I	Ethylene glycol	1.000	%(*v*/*v*)	V	Glycerol	72.0	%(*w*/*v*)
H9	9	Tris pH 8.0	0.15	*M*	J	Sodium potassium tartrate	0.010	*M*	W	Ammonium sulfate	2.8	*M*
H10	10	TAPS pH 8.5	0.15	*M*	K	MPD	1.000	%(*v*/*v*)	X	PEG 8000	40.0	%(*w*/*v*)
H11	11	AMPD/Tris pH 9.0	0.15	*M*	L	2-Butanol	1.000	%(*v*/*v*)	M	Phosphate	1.2	*M*
H12	12	CAPSO pH 9.5	0.15	*M*	A	Potassium bromide	0.020	*M*	N	PEG MME 550	48.00	%(*v*/*v*)

**Table 2:** Details of the samples used with the Pi minimal screen (Table 1 ▶) and number of crystallization hits TEN, buffer consisting of 20m*M* 2-amino-2-(hydroxymethyl)-1,3-propanediol (Tris), 1m*M* ethylenediaminetetraacetic acid (EDTA), 1m*M* sodium azide and 200m*M* sodium chloride.

Protein	Concentration (mgml^1^)	MW (kDa)	Source and code	Buffer/preparation	Hits
Lysozyme	10.0	14.4	Sigma L6876	Deionized water	56
Concanavalin A	7.0	26.5	Sigma L7647	TEN pH 8.5	16
Glucose isomerase	33.0	43.0	Hampton HR7-102	See product user guide	11
Xylanase	36.0	21.0	Hampton HR7-106	See product user guide	8
Ferritin	50150	440.0	Fluka 96701	As supplied by the manufacturer	8
Catalase	12.6	62.5	Sigma C3155	Deionized water	6
Citrate synthase	10.0	49.0	Sigma C3260	TEN pH 8.5	5
Lipase B	25.0	35.0	Hampton HR7-099	Deionized water	4
Ribonuclease A	30.0	13.7	Sigma R5503	Deionized water	1
Thaumatin	30.0	22.0	Sigma T7638	Deionized water	1
Sum					116

**Table 3:** ‘Hits per condition’ observed and corresponding results expected with the binomial distribution (Pi minimal screen)

Hits/condition	Observed	Expected
0	21	26.5
1	45	36.4
2	20	22.5
3 or more	10	10.6
Sum	96	96

**Table 4:** Final formulation of the Pi-PEG screen

	Set 1				Set 2				Set 3			
Well	ID	Name	Conc.	Unit	ID	Name	Conc.	Unit	ID	Name	Conc.	Unit
A1	1	Acetate pH 4.8	0.05	*M*	A	PEG 200	22.5	%(*v*/*v*)	M			
A2	2	Acetate pH 5.2	0.05	*M*	B	PEG 300	20.0	%(*v*/*v*)	N			
A3	3	MES pH 5.6	0.05	*M*	C	PEG MME 350	20.0	%(*v*/*v*)	O			
A4	4	MES pH 6.0	0.05	*M*	D	PEG 400	20.0	%(*v*/*v*)	P			
A5	5	ADA pH 6.4	0.05	*M*	E	PEG MME 550	20.0	%(*v*/*v*)	Q			
A6	6	ADA pH 6.8	0.05	*M*	F	PEG 600	20.0	%(*v*/*v*)	R			
A7	7	HEPES pH 7.1	0.05	*M*	G	PEG 1000	17.5	%(*w*/*v*)	S			
A8	8	HEPES pH 7.3	0.05	*M*	H	PEG 1500	17.5	%(*w*/*v*)	T			
A9	9	Tris pH 7.6	0.05	*M*	I	PEG 2000	15.0	%(*w*/*v*)	U			
A10	10	Tris pH 8.0	0.05	*M*	J	PEG MME 2000	15.0	%(*w*/*v*)	V			
A11	11	Bicine pH 8.4	0.05	*M*	K	PEG 3000	15.0	%(*w*/*v*)	W			
A12	12	Bicine pH 8.8	0.05	*M*	L	PEG 4000	15.0	%(*w*/*v*)	X			
B1	1	Acetate pH 4.8	0.05	*M*	H	PEG 1500	15.0	%(*w*/*v*)	O	PEG 4000	3.6	%(*w*/*v*)
B2	2	Acetate pH 5.2	0.05	*M*	I	PEG 2000	12.9	%(*w*/*v*)	P	PEG 200	6.4	%(*v*/*v*)
B3	3	MES pH 5.6	0.05	*M*	J	PEG MME 2000	12.9	%(*w*/*v*)	Q	PEG 300	5.7	%(*v*/*v*)
B4	4	MES pH 6.0	0.05	*M*	K	PEG 3000	12.9	%(*w*/*v*)	R	PEG MME 350	5.7	%(*v*/*v*)
B5	5	ADA pH 6.4	0.05	*M*	L	PEG 4000	12.9	%(*w*/*v*)	S	PEG 400	5.7	%(*v*/*v*)
B6	6	ADA pH 6.8	0.05	*M*	A	PEG 200	19.3	%(*v*/*v*)	T	PEG MME 550	5.7	%(*v*/*v*)
B7	7	HEPES pH 7.1	0.05	*M*	B	PEG 300	17.1	%(*v*/*v*)	U	PEG 600	5.7	%(*v*/*v*)
B8	8	HEPES pH 7.3	0.05	*M*	C	PEG MME 350	17.1	%(*v*/*v*)	V	PEG 1000	5.0	%(*w*/*v*)
B9	9	Tris pH 7.6	0.05	*M*	D	PEG 400	17.1	%(*v*/*v*)	W	PEG 1500	5.0	%(*w*/*v*)
B10	10	Tris pH 8.0	0.05	*M*	E	PEG MME 550	17.1	%(*v*/*v*)	X	PEG 2000	4.3	%(*w*/*v*)
B11	11	Bicine pH 8.4	0.05	*M*	F	PEG 600	17.1	%(*v*/*v*)	M	PEG MME 2000	4.3	%(*w*/*v*)
B12	12	Bicine pH 8.8	0.05	*M*	G	PEG 1000	15.0	%(*w*/*v*)	N	PEG 3000	4.3	%(*w*/*v*)
C1	1	Acetate pH 4.8	0.05	*M*	C	PEG MME 350	14.3	%(*v*/*v*)	Q	PEG 300	11.4	%(*v*/*v*)
C2	2	Acetate pH 5.2	0.05	*M*	D	PEG 400	14.3	%(*v*/*v*)	R	PEG MME 350	11.4	%(*v*/*v*)
C3	3	MES pH 5.6	0.05	*M*	E	PEG MME 550	14.3	%(*v*/*v*)	S	PEG 400	11.4	%(*v*/*v*)
C4	4	MES pH 6.0	0.05	*M*	F	PEG 600	14.3	%(*v*/*v*)	T	PEG MME 550	11.4	%(*v*/*v*)
C5	5	ADA pH 6.4	0.05	*M*	G	PEG 1000	12.5	%(*w*/*v*)	U	PEG 600	11.4	%(*v*/*v*)
C6	6	ADA pH 6.8	0.05	*M*	H	PEG 1500	12.5	%(*w*/*v*)	V	PEG 1000	10.0	%(*w*/*v*)
C7	7	HEPES pH 7.1	0.05	*M*	I	PEG 2000	10.7	%(*w*/*v*)	W	PEG 1500	10.0	%(*w*/*v*)
C8	8	HEPES pH 7.3	0.05	*M*	J	PEG MME 2000	10.7	%(*w*/*v*)	X	PEG 2000	8.6	%(*w*/*v*)
C9	9	Tris pH 7.6	0.05	*M*	K	PEG 3000	10.7	%(*w*/*v*)	M	PEG MME 2000	8.6	%(*w*/*v*)
C10	10	Tris pH 8.0	0.05	*M*	L	PEG 4000	10.7	%(*w*/*v*)	N	PEG 3000	8.6	%(*w*/*v*)
C11	11	Bicine pH 8.4	0.05	*M*	A	PEG 200	16.1	%(*v*/*v*)	O	PEG 4000	7.1	%(*w*/*v*)
C12	12	Bicine pH 8.8	0.05	*M*	B	PEG 300	14.3	%(*v*/*v*)	P	PEG 200	12.9	%(*v*/*v*)
D1	1	Acetate pH 4.8	0.05	*M*	J	PEG MME 2000	8.6	%(*w*/*v*)	S	PEG 400	17.1	%(*w*/*v*)
D2	2	Acetate pH 5.2	0.05	*M*	K	PEG 3000	8.6	%(*w*/*v*)	T	PEG MME 550	17.1	%(*v*/*v*)
D3	3	MES pH 5.6	0.05	*M*	L	PEG 4000	8.6	%(*w*/*v*)	U	PEG 600	17.1	%(*v*/*v*)
D4	4	MES pH 6.0	0.05	*M*	A	PEG 200	12.9	%(*v*/*v*)	V	PEG 1000	15.0	%(*w*/*v*)
D5	5	ADA pH 6.4	0.05	*M*	B	PEG 300	11.4	%(*v*/*v*)	W	PEG 1500	15.0	%(*w*/*v*)
D6	6	ADA pH 6.8	0.05	*M*	C	PEG MME 350	11.4	%(*v*/*v*)	X	PEG 2000	12.9	%(*w*/*v*)
D7	7	HEPES pH 7.1	0.05	*M*	D	PEG 400	11.4	%(*v*/*v*)	M	PEG MME 2000	12.9	%(*w*/*v*)
D8	8	HEPES pH 7.3	0.05	*M*	E	PEG MME 550	11.4	%(*v*/*v*)	N	PEG 3000	12.9	%(*w*/*v*)
D9	9	Tris pH 7.6	0.05	*M*	F	PEG 600	11.4	%(*v*/*v*)	O	PEG 4000	10.7	%(*w*/*v*)
D10	10	Tris pH 8.0	0.05	*M*	G	PEG 1000	10.0	%(*w*/*v*)	P	PEG 200	19.3	%(*v*/*v*)
D11	11	Bicine pH 8.4	0.05	*M*	H	PEG 1500	10.0	%(*w*/*v*)	Q	PEG 300	17.1	%(*v*/*v*)
D12	12	Bicine pH 8.8	0.05	*M*	I	PEG 2000	8.6	%(*w*/*v*)	R	PEG MME 350	17.1	%(*v*/*v*)
E1	1	Acetate pH 4.8	0.05	*M*	E	PEG MME 550	8.6	%(*v*/*v*)	U	PEG 600	22.9	%(*v*/*v*)
E2	2	Acetate pH 5.2	0.05	*M*	F	PEG 600	8.6	%(*v*/*v*)	V	PEG 1000	20.0	%(*w*/*v*)
E3	3	MES pH 5.6	0.05	*M*	G	PEG 1000	7.5	%(*w*/*v*)	W	PEG 1500	20.0	%(*w*/*v*)
E4	4	MES pH 6.0	0.05	*M*	H	PEG 1500	7.5	%(*w*/*v*)	X	PEG 2000	17.1	%(*w*/*v*)
E5	5	ADA pH 6.4	0.05	*M*	I	PEG 2000	6.4	%(*w*/*v*)	M	PEG MME 2000	17.1	%(*w*/*v*)
E6	6	ADA pH 6.8	0.05	*M*	J	PEG MME 2000	6.4	%(*w*/*v*)	N	PEG 3000	17.1	%(*w*/*v*)
E7	7	HEPES pH 7.1	0.05	*M*	K	PEG 3000	6.4	%(*w*/*v*)	O	PEG 4000	14.3	%(*w*/*v*)
E8	8	HEPES pH 7.3	0.05	*M*	L	PEG 4000	6.4	%(*w*/*v*)	P	PEG 200	25.7	%(*v*/*v*)
E9	9	Tris pH 7.6	0.05	*M*	A	PEG 200	9.6	%(*v*/*v*)	Q	PEG 300	22.9	%(*v*/*v*)
E10	10	Tris pH 8.0	0.05	*M*	B	PEG 300	8.6	%(*v*/*v*)	R	PEG MME 350	22.9	%(*v*/*v*)
E11	11	Bicine pH 8.4	0.05	*M*	C	PEG MME 350	8.6	%(*v*/*v*)	S	PEG 400	22.9	%(*v*/*v*)
E12	12	Bicine pH 8.8	0.05	*M*	D	PEG 400	8.6	%(*v*/*v*)	T	PEG MME 550	22.9	%(*v*/*v*)
F1	1	Acetate pH 4.8	0.05	*M*	L	PEG 4000	4.3	%(*w*/*v*)	W	PEG 1500	25.0	%(*w*/*v*)
F2	2	Acetate pH 5.2	0.05	*M*	A	PEG 200	6.4	%(*v*/*v*)	X	PEG 2000	21.4	%(*w*/*v*)
F3	3	MES pH 5.6	0.05	*M*	B	PEG 300	5.7	%(*v*/*v*)	M	PEG MME 2000	21.4	%(*w*/*v*)
F4	4	MES pH 6.0	0.05	*M*	C	PEG MME 350	5.7	%(*v*/*v*)	N	PEG 3000	21.4	%(*w*/*v*)
F5	5	ADA pH 6.4	0.05	*M*	D	PEG 400	5.7	%(*v*/*v*)	O	PEG 4000	17.9	%(*w*/*v*)
F6	6	ADA pH 6.8	0.05	*M*	E	PEG MME 550	5.7	%(*v*/*v*)	P	PEG 200	32.1	%(*v*/*v*)
F7	7	HEPES pH 7.1	0.05	*M*	F	PEG 600	5.7	%(*v*/*v*)	Q	PEG 300	28.6	%(*v*/*v*)
F8	8	HEPES pH 7.3	0.05	*M*	G	PEG 1000	5.0	%(*w*/*v*)	R	PEG MME 350	28.6	%(*v*/*v*)
F9	9	Tris pH 7.6	0.05	*M*	H	PEG 1500	5.0	%(*w*/*v*)	S	PEG 400	28.6	%(*v*/*v*)
F10	10	Tris pH 8.0	0.05	*M*	I	PEG 2000	4.3	%(*w*/*v*)	T	PEG MME 550	28.6	%(*v*/*v*)
F11	11	Bicine pH 8.4	0.05	*M*	J	PEG MME 2000	4.3	%(*w*/*v*)	U	PEG 600	28.6	%(*v*/*v*)
F12	12	Bicine pH 8.8	0.05	*M*	K	PEG 3000	4.3	%(*w*/*v*)	V	PEG 1000	25.0	%(*w*/*v*)
G1	1	Acetate pH 4.8	0.05	*M*	G	PEG 1000	2.5	%(*w*/*v*)	M	PEG MME 2000	25.7	%(*w*/*v*)
G2	2	Acetate pH 5.2	0.05	*M*	H	PEG 1500	2.5	%(*w*/*v*)	N	PEG 3000	25.7	%(*w*/*v*)
G3	3	MES pH 5.6	0.05	*M*	I	PEG 2000	2.1	%(*w*/*v*)	O	PEG 4000	21.4	%(*w*/*v*)
G4	4	MES pH 6.0	0.05	*M*	J	PEG MME 2000	2.1	%(*w*/*v*)	P	PEG 200	38.6	%(*v*/*v*)
G5	5	ADA pH 6.4	0.05	*M*	K	PEG 3000	2.1	%(*w*/*v*)	Q	PEG 300	34.3	%(*v*/*v*)
G6	6	ADA pH 6.8	0.05	*M*	L	PEG 4000	2.1	%(*w*/*v*)	R	PEG MME 350	34.3	%(*v*/*v*)
G7	7	HEPES pH 7.1	0.05	*M*	A	PEG 200	3.2	%(*v*/*v*)	S	PEG 400	34.3	%(*v*/*v*)
G8	8	HEPES pH 7.3	0.05	*M*	B	PEG 300	2.9	%(*v*/*v*)	T	PEG MME 550	34.3	%(*v*/*v*)
G9	9	Tris pH 7.6	0.05	*M*	C	PEG MME 350	2.9	%(*v*/*v*)	U	PEG 600	34.3	%(*v*/*v*)
G10	10	Tris pH 8.0	0.05	*M*	D	PEG 400	2.9	%(*v*/*v*)	V	PEG 1000	30.0	%(*w*/*v*)
G11	11	Bicine pH 8.4	0.05	*M*	E	PEG MME 550	2.9	%(*v*/*v*)	W	PEG 1500	30.0	%(*w*/*v*)
G12	12	Bicine pH 8.8	0.05	*M*	F	PEG 600	2.9	%(*v*/*v*)	X	PEG 2000	25.7	%(*w*/*v*)
H1	1	Acetate pH 4.8	0.05	*M*	B				O	PEG 4000	25.0	%(*w*/*v*)
H2	2	Acetate pH 5.2	0.05	*M*	C				P	PEG 200	45.0	%(*v*/*v*)
H3	3	MES pH 5.6	0.05	*M*	D				Q	PEG 300	40.0	%(*v*/*v*)
H4	4	MES pH 6.0	0.05	*M*	E				R	PEG MME 350	40.0	%(*v*/*v*)
H5	5	ADA pH 6.4	0.05	*M*	F				S	PEG 400	40.0	%(*v*/*v*)
H6	6	ADA pH 6.8	0.05	*M*	G				T	PEG MME 550	40.0	%(*v*/*v*)
H7	7	HEPES pH 7.1	0.05	*M*	H				U	PEG 600	40.0	%(*v*/*v*)
H8	8	HEPES pH 7.3	0.05	*M*	I				V	PEG 1000	35.0	%(*w*/*v*)
H9	9	Tris pH 7.6	0.05	*M*	J				W	PEG 1500	35.0	%(*w*/*v*)
H10	10	Tris pH 8.0	0.05	*M*	K				X	PEG 2000	30.0	%(*w*/*v*)
H11	11	Bicine pH 8.4	0.05	*M*	L				M	PEG MME 2000	30.0	%(*w*/*v*)
H12	12	Bicine pH 8.8	0.05	*M*	A				N	PEG 3000	30.0	%(*w*/*v*)
